# Narrow-Band Imaging Magnifying Endoscopy versus Lugol Chromoendoscopy with Pink-Color Sign Assessment in the Diagnosis of Superficial Esophageal Squamous Neoplasms: A Randomised Noninferiority Trial

**DOI:** 10.1155/2015/639462

**Published:** 2015-07-01

**Authors:** Kenichi Goda, Akira Dobashi, Noboru Yoshimura, Masayuki Kato, Hiroyuki Aihara, Kazuki Sumiyama, Hirobumi Toyoizumi, Tomohiro Kato, Masahiro Ikegami, Hisao Tajiri

**Affiliations:** ^1^Department of Endoscopy, The Jikei University School of Medicine, 3-25-8 Nishi-Shinbashi, Minato-ku, Tokyo 105-8461, Japan; ^2^Department of Pathology, The Jikei University School of Medicine, 3-25-8 Nishi-Shinbashi, Minato-ku, Tokyo 105-8461, Japan

## Abstract

Previous studies have shown the high diagnostic accuracy of narrow-band imaging magnifying endoscopy (NBI-ME) and Lugol chromoendoscopy with pink-color sign assessment (LCE-PS) for superficial esophageal squamous cell carcinoma (SESCC). However, there has been no controlled trial comparing these two diagnostic techniques. We conducted a randomized noninferiority trial to compare the diagnostic accuracy of NBI-ME and LCE-PS. We recruited patients with, or with a history of, squamous cell carcinoma in the head and neck region or in the esophagus. They were randomly assigned to either NBI-ME or LCE-PS. When lesions > 5 mm in diameter were found as brownish areas on NBI or as Lugol-voiding lesions (LVL), they were evaluated to determine whether they are SESCC on the basis of the findings of NBI-ME or PS in the LVL. NBI-ME and LCE-PS were completed in 147 patients each. There was no significant difference in all diagnostic values between the two techniques. Compared with LCE-PS, NBI-ME showed a significantly shorter examination time but a larger number of misdiagnosed lesions especially in patients with many irregularly shaped multiform LVLs. Compared with LCE-PS, NBI-ME might be similarly accurate and less invasive, but less reliable in patients with many LVLs, in the diagnosis of SESCC.

## 1. Introduction

Esophageal cancer accounted for 407,000 deaths, with 482,000 new cases, in 2008. It ranks as the eighth most common cancer worldwide and the sixth most common cause of death from cancer [1]. Esophageal cancers are histologically classified as squamous cell carcinoma (SCC) or adenocarcinoma [2]. About 86% of deaths due to esophageal cancer occur in developing countries, and most of the cases (90%) are esophageal SCC (ESCC) [1]. The highest mortality rates are found in eastern and southern Africa and in eastern Asia including China and Japan [3].

ESCC patients have a dismal prognosis because >50% of them already have advanced-stage cancer with unresectable and/or metastatic disease at manifestation [4]. Patients with superficial ESCC (SESCC), in which the infiltration is confined to the mucosa or submucosa, have a considerably better prognosis than those with advanced-stage ESCC [5]. However, early detection of ESCC is difficult because SESCC often shows a flat and/or isochromatic lesion on conventional white light imaging endoscopy (CWE) [6, 7].

Lugol chromoendoscopy (LCE) is the worldwide gold standard for detecting superficial ESCC [8–10]. LCE can visualize superficial squamous neoplasms as a Lugol-voiding lesion (LVL), manifesting as an iodine-unstained area, even if the neoplasms were invisible on CWE. LCE, however, occasionally causes heartburn and severe discomfort, has a risk of causing allergic reaction, and increases the duration of endoscopic examination [10, 11]. Moreover, esophageal LVLs include a wide variety of histologies ranging from high-grade neoplasia such as ESCC to not only low-grade neoplasia but also nonneoplasia such as inflammation and a normal mucosa [12]. Hence, LCE is a highly sensitive but not very specific technique for detecting SESCC.

A previous study suggested that the diagnostic accuracy of LCE for SESCC detection was remarkably improved by additionally assessing for a reddish or rose-pink color change, the so-called pink-color sign (PS), in an LVL. LCE plus PS assessment (LCE-PS) showed considerably high sensitivity and specificity of 91.9% and 94%, respectively [13].

Narrow-band imaging (NBI) is a revolutionary technology of optical image-enhanced endoscopy that was first described in 2004 [14]. The details of the NBI system have been published elsewhere [15].

Previous studies on superficial squamous neoplasms in the orohypopharynx and esophagus showed that the NBI technology visualized subtle lesions of superficial squamous neoplasms as a well-demarcated brownish area without Lugol's iodine staining [16, 17].

In addition, NBI combined with magnifying endoscopy (NBI-ME) can distinctly visualize the capillary microvasculature of the mucosal surface of the esophagus (i.e., intrapapillary capillary loop (IPCL)) [18]. Previous studies demonstrated that NBI-ME was useful in predicting the histology of SESCC by evaluating the morphologic change of the IPCL in detail [6, 7, 18, 19]. A multicenter randomized control trial indicated that NBI-ME had a significantly higher detection rate than CWE (97.2% versus 55.2%); however, the median examination time of NBI-ME was significantly longer than that of CWE [20]. Other than the longer examination time, there are no reports about adverse effects caused by NBI-ME, whereas LCE sometimes causes adverse conditions as mentioned above.

There seems to be a lack of investigations comparing the diagnostic performance of NBI and LCE for SESCC detection. A study showed that nonmagnified endoscopy with NBI was significantly superior to LCE (not LCE-PS) in terms of positive predictive value (4.4% versus 9.8%) [21]. Takenaka et al. reported that NBI-ME can detect SESCCs with lower sensitivity, but significantly higher specificity and overall accuracy, than LCE (not LCE-PS) [22].

To our best knowledge, however, there is no study comparing the diagnostic accuracies of NBI-ME and LCE-PS for SESCC. Accordingly, we conducted the present randomized noninferiority trial to compare the two techniques.

## 2. Materials and Methods

### 2.1. Rationale

#### 2.1.1. Study Design and Sample Size

Both NBI-ME and LCE-PS were reported to have high sensitivities for SESCC, at 97.2% and 91.9%, respectively [13, 20]. We considered that these two endoscopic inspection techniques have a clinically high enough diagnostic accuracy for SESCC. As mentioned before, NBI-ME has some clinical advantages over LCE-PS, which could produce adverse effects in patients. We aimed to verify the clinical utility of NBI-ME, although it is not superior to LCE-PS, by finding a statistically significant difference in their diagnostic abilities. Therefore, we designed this controlled study as a noninferiority test.

We calculated the required sample size for a two-armed noninferiority test according to a statistics textbook [23]. Considering LCE-PS as reference endoscopy technique, baseline sensitivity was assumed as 0.919 (i.e., 91.9%) from a previous study [13]. A sensitivity of 0.8 (i.e., 80%) would be a clinically adequate diagnostic value for screening or surveillance endoscopy for SESCC. Therefore, assuming an equivalence difference of −0.1 and an actual difference of 0, the calculated sample size was 290 (145 per group) for an *α* value of 0.05 and *β* value of 0.1. Assuming a dropout or ineligibility rate of 5%, at least 305 subjects seemed to be required for the eligibility assessment.

#### 2.1.2. Study Population

A previous study described that the incidence rate of synchronous SESCC in patients with head and neck SCC (HNSCC) was 13.9% and that of metachronous SESCC was 3.0% [24]. Two other studies showed that the incidence rate of synchronous or metachronous SESCC was 10% to 14.6% in patients with SESCC who had been treated with endoscopic resection during an endoscopic follow-up of 12 months or longer [25, 26]. Hence, patients with a history of HNSCC or ESCC were considered as good candidates for a screening or surveillance endoscopy model like the present study.

#### 2.1.3. Target Lesions

SESCC lesions are sometimes difficult to detect by CWE alone, in contrast to massively invasive SCC lesions such as an advanced cancer. We targeted only SESCC, defined as SCC invading up to the submucosa and high-grade intraepithelial neoplasia (HGIN).

The mechanism underlying the carcinogenesis of ESCC has been proposed to be a dysplasia-carcinoma sequence, progressing from mild or moderate dysplasia (low-grade dysplasia) to severe dysplasia (high-grade dysplasia) and SCC [27]. On the basis of previous studies, the World Health Organization proposed that detection of HGIN including high-grade dysplasia and SCC* in situ* is clinically important because HGINs have a considerable potential to become malignant invasive cancers [27–29]. Consequently, we included HGIN in the histological definition of SESCC in the present study.

### 2.2. Participants

We conducted a prospective randomized controlled study at Jikei University Hospital from January 2009 to June 2011. This study was performed in accordance with the Declaration of Helsinki. The institutional review board approved the study protocol. Written informed consent was obtained from all participants.

Participants were considered eligible if they met all of the following inclusion criteria: (i) histologically confirmed HNSCC or a history of HNSCC or ESCC; (ii) age of 20 years or older; and (iii) no symptom of dysphagia. If advanced ESCCs without severe stricture were found in the enrolled patients, we did not evaluate primary advanced cancers but concomitant SESCC.

Patients were excluded if any one of the following exclusion criteria was met: (i) previous esophageal surgery; (ii) history of chemoradiotherapy or radiotherapy for ESCC; (iii) recent history of chemotherapy for any malignancy; (iv) history of intolerance to Lugol chromoendoscopy or allergic reaction to iodine; (v) concurrent presentation of an esophageal varix; (vi) current pregnancy in women; and (vii) prohibition against stopping antiplatelet or anticoagulant medication.

### 2.3. Interventions and Masking (Randomization)

Participants were randomly assigned at a 1 : 1 ratio to either the NBI-ME or LCE-PS group. Randomization using numbered sealed envelopes was carried out by an office administrator of our department who is not directly involved in the present study.

A randomly assigned endoscopic inspection was performed by one of the two expert endoscopists (Kenichi Goda or Noboru Yoshimura). Both of the endoscopists were not aware of any information on past endoscopic findings and histology. The other endoscopist (Akira Dobashi) checked the medical records of all participants and managed the randomization to the endoscopic procedures, as well as filling out all case report forms (CRFs).

### 2.4. Standardization of the Endoscopic Diagnosis

**Figure 1:**
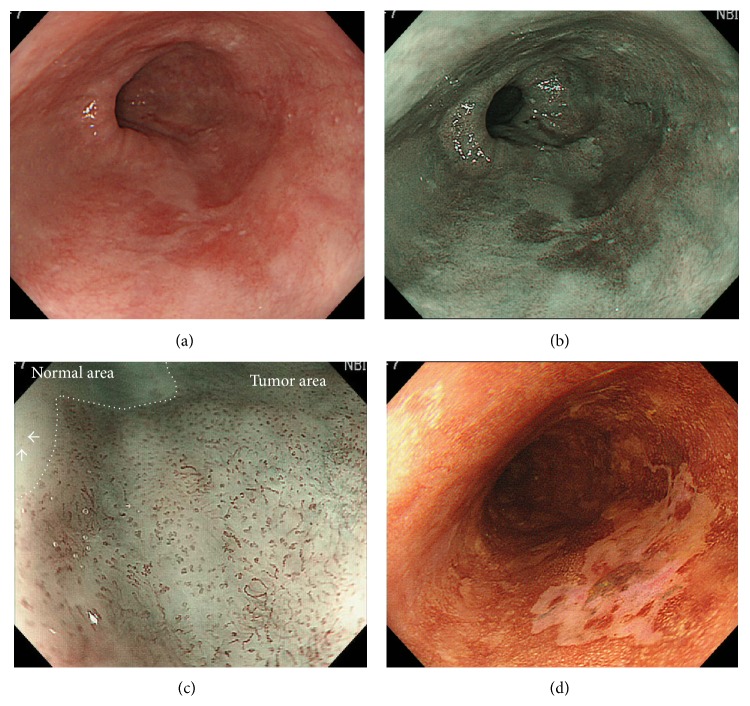
A representative lesion of superficial esophageal squamous cell carcinoma. (a) A flat-type lesion of reddish color could be observed at the two to seven o'clock position in the middle esophagus. (b) The lesion was clearly visualized as a 30 mm wide brownish area by using nonmagnifying endoscopy with narrow-band imaging. (c) Narrow-band imaging magnifying endoscopy shows an intervascular background coloration and increase in the number of abnormal microvessels (i.e., proliferation) in the tumor area compared with the normal area. Abnormal microvessels with morphological changes of dilation, tortuosity, change in caliber, and various shapes compared with normal intrapapillary capillary loops are also seen (white arrows). (d) On Lugol chromoendoscopy, the lesion is visualized as a Lugol-voiding area with a pink-color sign at 3 min after spraying the iodine solution. The lesion was removed by endoscopic submucosal dissection, and the histology was squamous cell carcinoma invading up to the lamina propria mucosae.

The endoscopic procedures and diagnosis were all performed by two expert endoscopists (Kenichi Goda and Noboru Yoshimura) who both had experiences in >200 NBI-ME and LCE-PS inspections for SESCC cases. Immediately before the present study, the two endoscopists reviewed typical still images of NBI-ME and LCE-PS that were taken from 20 SESCC lesions and 10 nonneoplastic lesions comprising 4 inflammatory lesions, 3 papillomas, and 3 normal squamous epitheliums. The 20 SESCC lesions were all visualized as a well-demarcated brownish area with “NBI-ME positive” abnormal IPCLs mentioned below and as an LVL with PS on LCE. One representative case out of the 20 SESCC lesions is shown in Figures 1(a)–1(d).

### 2.5. Procedures and Endoscopy Systems

All patients were orally administered with 20,000 U Pronase (Pronase MS; Kaken Pharmaceutical Products Inc., Tokyo, Japan) before the administration of pharyngeal anesthesia to eliminate mucus in the esophagus. All endoscopic inspections were performed under deep sedation through intravenous administration of pethidine hydrochloride (35–70 mg, Opystan; Mitsubishi Tanabe Pharma, Osaka, Japan) and flunitrazepam (0.2–0.8 mg, Rohypnol; Chugai Pharmaceutical, Tokyo, Japan).

NBI-ME and LCE-PS were performed by using a high-definition zoom endoscope (GIF-H260Z; Olympus Co., Tokyo, Japan) and a 19-in high-resolution liquid-crystal monitor (OEV191H; Olympus Co.) that enabled endoscopic observation at a 90-fold maximum magnification. A black rubber attachment (MB-46, Olympus Co.) was mounted on the tip of the zoom endoscope to maintain the focal distance between the tip of the scope and the lesion surface at 2 mm, and it facilitated precise focusing during the magnification observation.

### 2.6. Endoscopic Evaluation and Biopsy Protocol

Endoscopic inspection with NBI-ME or LCE-PS was started from a point about 20 cm from the upper incisors in the cervical esophagus toward the esophagogastric junction, and then the inspection was finished when the endoscope was withdrawn up to the same point in the cervical esophagus. We recorded the examination time of the reciprocating observation for the esophagus because we routinely observe the esophagus during both the processes of insertion and withdrawal of the endoscope. The examination time does not include the biopsy procedure time.

First, we selected a well-demarcated brownish area on NBI or an LVL on LCE when it has a diameter of >5 mm. The diameter of each area was estimated by comparing it with the width of the biopsy forceps (Radial Jaw 3; Boston Scientific, Natick, MA, USA) when opened, which was approximately 6 mm.

Next, we focused on six NBI-ME findings in the brownish area (the definitions and schemas for which are listed in Table 1) and evaluated the presence or absence of each NBI-ME finding. The brownish area was evaluated as “NBI-ME positive” for SESCC when it had at least four of the six abnormal MBI-ME findings that were mentioned in previous studies [19, 30, 31].

**Figure 2:**
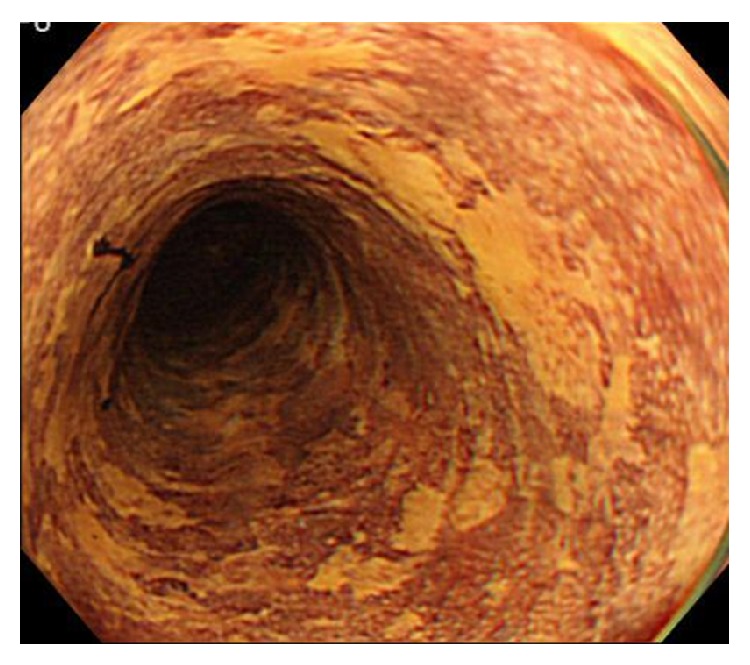
Lugol chromoendoscopy image of many irregularly shaped multiform Lugol-voiding lesions (grade D Lugol-voiding pattern).

On LCE, the presence or absence of PS in the LVL was evaluated 3 min after spraying with a Lugol dye solution [13]. The LVL was evaluated as “LCE-PS positive” for SESCC when PS appeared in the LVL. In addition, Muto et al. [24] classified the grades of the Lugol-voiding pattern in the background esophageal mucosa on the basis of the number, diameter, and shape of the LVL as follows: grade A, no LVLs; B, several (≤10) small LVLs; C, many (>10) small LVLs; and D, many (>10) irregularly shaped multiform LVLs. LVLs < 5 mm in diameter were defined as small. LVLs > 5 mm in diameter and having irregular rims were defined as irregularly shaped multiform LVLs (grade D: Figure 2).

The macroscopic type of the tumor was determined according to the Paris classification [31]. Endoscopic findings and examination time were all recorded on the CRF for each patient.

### 2.7. Endpoints

The primary aim of this study is to compare the sensitivity of NBI-ME with that of LCE-PS in detecting SESCC. The secondary endpoints were to compare the other diagnostic accuracy measures (i.e., specificity, positive predictive value, negative predictive value, and overall accuracy), clinicopathological characteristics of misdiagnosed lesions, and examination time between NBI-ME and LCE-PS.

### 2.8. Histological Evaluation

The histology of biopsied or endoscopically/surgically resected specimens was established by a single pathologist (Masahiro Ikegami) who has expertise in gastrointestinal cancer according to the Japanese classification of esophageal cancer [32]. SESCC was histologically defined as HGIN or SCC invading up to the submucosa.

The pathologist was not aware of any endoscopic findings. If the histology results of a lesion in a patient were different between the biopsied and resected specimens, the worse histology was adopted as the final histology of the lesion and of the patient. If a patient had multiple lesions that had been biopsied or endoscopically/surgically removed, the worst histology was adopted as the final histology of the patient.

The pathologist evaluated the invasion depth of SESCCs from the resected specimens according to the Japanese classification of esophageal cancer as follows: T1a, tumor invading the mucosa (T1a-EP, carcinoma* in situ*; T1a-LPM, tumor invading the lamina propria mucosa; T1a-MM, tumor invading the muscularis mucosa); T1b, tumor invading the submucosa (SM1, invading to a depth of ≤200 *μ*m from the muscularis mucosa; SM2, extending > 200 *μ*m) [32].

### 2.9. Statistical Analysis

The histology of the biopsied and resected specimens was used as the gold standard for the diagnosis. Diagnostic accuracy measures such as sensitivity, specificity, positive predictive value, negative predictive value, and overall accuracy were calculated not on a lesion basis but on a patient basis because no tissue sample was biopsied from a mucosal site that did not show a brownish area or an LVL. A patient without a brownish area or an LVL was assumed to have a normal esophagus. Such cases were assumed to be non-SESCC negative for both NBI-ME and LCE-PS in the patient-based analysis. In addition, if a patient had multiple lesions, the worst histology was used for the patient-based analysis. The examination times needed for NBI-ME or LCE-PS were statistically compared by using the median values.

Quantitative parameters were compared with Student's *t*-test or Mann-Whitney *U* test, and qualitative parameters were compared by using Pearson's *χ* ^2^ test. Statistical significance was accepted for *P* values of 0.05.

## 3. Results

**Figure 3:**
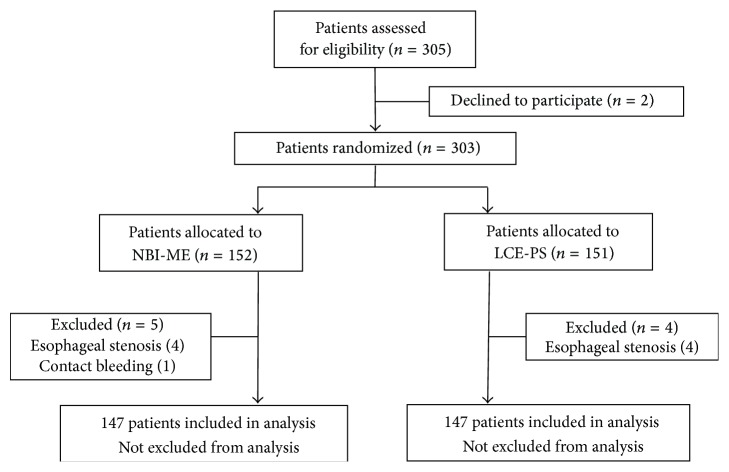
Overview of the study design.* NBI-ME*: narrow-band imaging magnifying endoscopy;* LCE-PS*: Lugol chromoendoscopy with pink-color sign assessment.

An overview of the workflow of this study is shown in Figure 3. We recruited 305 patients who met the inclusion criteria from January 2010 to June 2011. Two subjects declined to participate in the study. Three hundred and three patients were enrolled, and 151 and 152 patients were randomly assigned into the NBI-ME and LCE-PS groups, respectively.

A total of nine patients were excluded from the analysis, five from the NBI-ME group and four from the LCE-PS group. Eight of the nine patients had esophageal stenosis, due to scar formation after a previous endoscopic resection in four patients and due to advanced ESCC in the other four patients. Although all of the eight patients had no symptoms such as dysphasia, a high-definition magnifying endoscope could not pass the stenotic areas. In the remaining one of the nine patients, NBI-ME had to be stopped because of a continuous oozing bleeding from a protruding-type ESCC lesion.

We completed NBI-ME in 147 patients and LCE-PS in another 147 patients. A total of 294 patients were included in the final analysis.

The patient demographics and SESCC lesion characteristics are listed in Table 2. There were no significant differences in all values of patient demographics and SESCC lesion characteristics between NBI-ME and LCE-PS. Twenty-two percent and 20% of the patients who underwent NBI-ME and LCE-PS, respectively, had many irregularly shaped multiform LVLs, that is, grade D Lugol-voiding pattern. Fifty-four and 62 SESCC lesions in 45 and 41 patients were detected with NBI-ME and LCE-PS, respectively. Forty-eight (88%) and 59 (95%) of 54 and 62 SESCCs that were detected with NBI-ME and LCE-PS, respectively, showed a type 0-II (superficial and flat-type) morphology. Forty-nine (91%) and 57 (88%) of 54 and 62 SESCCs detected with NBI-ME and LCE-PS, respectively, were histologically diagnosed as an HGIN or T1a tumor. Endoscopic resection was performed for approximately two-thirds of SESCCs detected with either NBI-ME or LCE-PS.

The relations between endoscopic diagnosis and final histology according to the patient-based analysis are shown in Table 3. Eight patients with SESCC lesions tested false negative for NBI-ME and eight patients had false-negative results on LCE-PS. Many irregularly shaped multiform LVLs (i.e., grade D Lugol-voiding pattern) were present in six (75%) and three (38%) patients with SESCC lesions negative for NBI-ME and LCE-PS, respectively.

A comparison of diagnostic accuracy measures between NBI-ME and LCE-PS is given in Table 4. In the per-patient analysis, the sensitivity, specificity, positive predictive value, negative predictive value, and overall accuracy of NBI-ME and LCE-PS for diagnosing SESCC were 82.2% and 80.5%, 95.1% and 94.3%, 88.1% and 84.6%, 92.4% and 92.6%, and 91.2% and 90.5%, respectively. There were no significant differences in all diagnostic accuracy measures between NBI-ME and LCE-PS.

The clinicopathological characteristics of misdiagnosed lesions are summarized in Table 5. The misdiagnosed lesions are divided into false-negative SESCCs and false-positive non-SESCC lesions and then subdivided into the SESCCs and the lesions detected with NBI-ME and LCE-PS. The total numbers of SESCCs or non-SESCC lesions misdiagnosed with NBI-ME and LCE-PS are 34 and 28, respectively. The median diameters of the misdiagnosed lesions are 10 or 12 mm. Most of the misdiagnosed lesions showed a macroscopic type of 0-IIb (i.e., completely flat). False-negative SESCCs for NBI-ME involved two tumors with an invasion deeper than the lamina propria mucosae (T1a-MM, 1; T1b-SM1, 1). Of the biopsied specimens, 87% and 63% of false-positive lesions showed a histology of low-grade intraepithelial neoplasia on both NBI-ME and LCE-PS. False-positive lesions for LCE-PS involved four inflammatory lesions (e.g., inflammation or necrosis), while those false positive for NBI-ME involved only one inflammatory lesion. False-positive SESCCs and false-positive lesions for NBI-ME tended to be accompanied with a background mucosa with a grade D Lugol-voiding pattern at higher proportions than those for LCE-PS (89% and 60% versus 67% and 44%, resp.).

Table 6 shows a comparison of the examination times between NBI-ME and LCE-PS. The median examination times (ranges) of NBI-ME and LCE-PS were 234 s (92–1459 s) and 349 s (246–655 s), respectively. The median examination time of NBI-ME was significantly shorter than that of LCE-PS (*P* < 0.001).

## 4. Discussion

The present study showed no significant differences between NBI-ME and LCE-ME in sensitivity and in any other diagnostic accuracy measure for SESCC detection. The sensitivity and overall accuracy of NBI-ME and LCE-PS were higher than 80% and 90%, respectively. Most of the SESCC lesions detected with NBI-ME or LCE-PS were superficial and flat-type tumors at a very early stage (HGIN or T1a). Therefore, NBI-ME and LCE-PS would both be useful techniques for the detection and characterization of SESCCs.

The sensitivity of NBI-ME in the present study was, however, lower than the previously reported sensitivities of 90.9% and 97.2% [20, 22]. Between the two studies, one defined the diagnostic criteria of NBI-ME for SESCC as two abnormal IPCL patterns (dilated and tortuous) in a well-demarcated brownish area, whereas the other study only used an irregular microvascular pattern as the diagnostic criteria. We defined the NBI-ME diagnostic criteria for SESCC by using at least four of six abnormal NBI-ME findings in a well-demarcated area, as shown in Table 1. The rigorous NBI-ME criteria for SESCC may have led to the lower sensitivity of NBI-ME in the present study.

As mentioned before, LCE has several drawbacks that have never been mentioned in studies with NBI-ME [10, 11]. Moreover, recent studies showed the utility of NBI-ME for the early detection of orohypopharyngeal SCC [16, 17, 20]. In the head and neck region, the orohypopharynx is the most common site where SCC is associated with ESCC synchronously or metachronously [33, 34]. In practical endoscopy, however, LCE is not applicable for detecting orohypopharyngeal SCC because spraying Lugol solution can cause aspiration [33, 34]. Thus, although the present study showed no significant difference in all diagnostic accuracy measures for SESCC between NBI-ME and LCE-PS, we suggest that NBI-ME has a substantial clinical advantage over LCE-PS.

Nevertheless, this study showed a larger number of SESCCs or lesions misdiagnosed by NBI-ME compared with LCE-PS. Two of the SESCC lesions that were false negative on NBI-ME were T1a-MM and T1b-SM1 tumors that have an unignorable risk for lymph-node metastasis (12.2% and 26.5%, resp.) [35]. The SESCCs or lesions misdiagnosed with NBI-ME tended to be more frequently accompanied by a background esophageal mucosa showing a grade D Lugol-voiding pattern than those misdiagnosed with LCE-PS. These results suggest that LCE-PS might be a more reliable detection tool than NBI-ME in cases with a background esophageal mucosa having a grade D Lugol-voiding pattern. In addition, it is suggested that LCE-PS should be added to NBI-ME in cases with a background esophageal mucosa having grade D Lugol-voiding pattern to reduce a risk of misdiagnosis.

Several studies suggested that an increased number of LVLs carry a greater risk of metachronous development of SCC in the head and neck as well as in the esophagus [24, 26, 36]. Therefore, LCE might be recommended as the initial endoscopic examination when the patient has a grade D LVL pattern (many irregularly shaped multiform LVLs) to assess the patient's risk of developing metachronous SCC in the head and neck and the esophagus.

The median examination time of LCE-PS was about 1.5 times as long as that of NBI-ME in the present study. This result could be related to the considerable time of the staining (Lugol's iodine) procedure and the 3 min wait time for assessing PS in LVL. Moreover, the LCE procedure needs preparation time for the Lugol solution and the spraying tube. NBI-ME might be less invasive for patients and less laborious for endoscopists than LCE-PS.

The number of biopsies required for detecting a SESCC lesion based upon brownish area alone on NBI without magnifying assessment is significantly smaller than that based upon LVL alone without PS assessment (1.7 versus 4.4: *P* < 0.001) in ad hoc analysis of the present study. Although this is not an endpoint of the present study, this result may suggest that NBI can reduce the number of biopsies in practical endoscopy for patients with HNSCC or ESCC. In addition, as mentioned before, LCE procedure involves costs of the Lugol solution and the spraying tube while NBI does not incur additional cost after purchase of the NBI endoscopy system. Hence, NBI will offer a better cost benefit compared to LCE.

The present study has several limitations. First, it was conducted in a single center and only by experts. A further study with a multicenter setting and involving nonexperts is needed to validate the results of the present study. Second, the population of patients who underwent NBI-ME was different from those who underwent LCE-PS because the present study was designed as a two-armed trial. Ideally, it would be favorable to compare the diagnostic accuracy of two different examinations in a single patient population. Third, the diagnostic values in the present study were calculated only according to a per-patient basis. However, unless a mucosal site that is endoscopically normal could be biopsied, a per-lesion analysis of diagnostic accuracy measures could not be performed.

## 5. Conclusions

The present study suggests that NBI-ME is a similarly accurate and less invasive technique for detecting SESCC compared with LCE-PS. LCE-PS might be more reliable than NBI-ME in detecting SESCC in patients with many irregularly shaped multiform LVLs.

## Figures and Tables

**Table 1:** Definitions and schemas of normal and abnormal microvessels.

Finding of NBI magnifying endoscopy	Definition	Schema
Normal	Superficial microvessel with a single loop but no changes in caliber or various shapes in the normal whitish mucosa (i.e., intraepithelial capillary loop (IPCL))	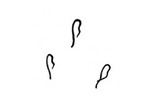
Abnormal		
Intervascular background coloration	Brownish coloration between microvessels which differed from whitish epithelium of surrounding normal mucosa	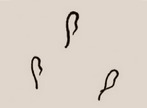
Proliferation	The presence of a group of higher dense microvessels compared with a density of IPCLs on surrounding normal mucosa	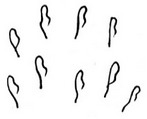
Dilation	Diameters of a group of microvessels which were at least twice compared with those of IPCLs on surrounding normal mucosa	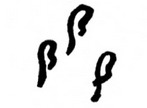
Tortuosity	The presence of a group of microvessels which are more greatly or sharply twisted or bent compared with IPCLs on surrounding normal mucosa	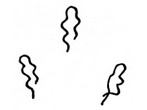
Changes in caliber	The presence of abrupt changes in vessel diameter (i.e., thickening or narrowing) in a group of microvessels	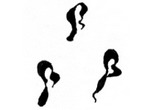
Various shapes	The presence of highly diverse morphologies in a group of microvessels	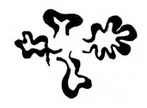

**Table 2:** Patient demographics and SESCC lesion characteristics.

	NBI-ME (*n* = 147)	LCE-PS (*n* = 147)	*P*
Patient demographics			
Age, years; median (range)	67 (39–86)	66 (35–85)	NS^†^
Men; *n*	130	131	NS^*∗*^
History of HNSCC/ESCC;			
Number of patients; *n*/*n*	85/74	88/69	NS^*∗*^
Drinking habit			
Number of drinkers; *n*	134	129	NS^*∗*^
Drinking duration, years; median (range)	40 (10–68)	40 (1–60)	NS^†^
Number of flushers; *n*	102	100	NS^*∗*^
Smoking habit			
Number of smokers; *n*	129	128	NS^*∗*^
Smoking, years; median (range)	35 (3–70)	37 (1–59)	NS^†^
Number of patients with grade D LVL pattern; *n*	33 (22%)	29 (20%)	NS^*∗*^
Final histology			
Number of patients with nonneoplasia/LGIN/SESCC	80/22/45	87/19/41	NS^*∗*^
SESCC lesion characteristics			
Number of SESCC lesions	54	62	NS^†^
Diameter, mm; median (range)	29 (4–100)	25 (6–70)	NS^†^
Macroscopic tumor type (0-I/IIa, IIb, and IIc/III)	5/48 (88%)/1	3/59 (95%)/0	NS^*∗*^
Histology, HGIN or T1a/T1b; *n*/*n*	49 (91%)/5	57 (88%)/5	NS^*∗*^
Treatment			
ER/SR/CRT/others	32/11/5/6	40/13/1/8	NS^*∗*^

NBI-ME: narrow-band imaging magnifying endoscopy; LCE-PS: Lugol chromoendoscopy with pink-color sign assessment; LVL: Lugol voiding lesion; LGIN: low-grade intraepithelial neoplasia; HGIN: high-grade intraepithelial neoplasia; SESCC: superficial esophageal squamous cell carcinoma. ER: endoscopic resection; SR: surgical resection; CRT: chemoradiotherapy; ^*∗*^Mann-Whitney *U* test; ^†^ *t*-test; NS: not significant.

**Table 3:** Relations between endoscopic diagnosis and final histology.

	SESCC	Nonneoplasia/LGIN
NBI-ME		
Positive	37 (11)	5 (1)
Negative	8 (6)	97 (15)
LCE-PS		
Positive	33 (11)	6 (2)
Negative	8 (3)	100 (14)

NBI-ME: narrow-band imaging magnifying endoscopy; LCE-PS: Lugol chromoendoscopy with pink-color sign assessment; LGIN: low-grade intraepithelial neoplasia; SESCC: superficial esophageal squamous cell carcinoma; (): number of patients with grade D Lugol voiding pattern.

**Table 4:** Comparison of diagnostic outcomes between NBI-ME and LCE-PS.

	NBI-ME	LCE-PS	*P*
Sensitivity, % (95% CI)	82.2 (67.9–92.0)	80.5 (65.1–91.2)	NS^*∗*^
Specificity, % (95% CI)	95.1 (88.9–98.4)	94.3 (88.1–97.9)	NS^*∗*^
PPV, % (95% CI)	88.1 (74.4–96.0)	84.6 (69.5–94.1)	NS^*∗*^
NPV, % (95% CI)	92.4 (85.5–96.7)	92.6 (85.9–96.7)	NS^*∗*^
Overall accuracy, % (95% CI)	91.2 (85.4–95.2)	90.5 (84.5–94.7)	NS^*∗*^

NBI-ME, narrow-band imaging magnifying endoscopy; LCE-PS, Lugol chromoendoscopy with pink-color sign assessment; LGIN, low-grade intraepithelial neoplasia; SESCC, superficial esophageal squamous cell carcinoma; CI, confidence interval; PPV, positive predictive value; NPV, negative predictive value; NS, not significant; ^*∗*^Pearson's *χ* ^2^ test.

**Table 5:** Clinicopathological characteristics of misdiagnosed lesions.

	False-negative SESCCs		False-positive non-SESCC lesions	
	NBI-ME	LCE-PS	NBI-ME	LCE-PS
Number of lesions	19	12	15	16
Diameter, mm; median (range)	12 (4–35)	10 (6–25)	12 (6–30)	10 (6–40)
Macroscopic type, *n* (0-I/IIa, IIb, IIc/III)	0/0, 17, 2/0	0/2, 10, 0/0	0/1, 12, 2/0	0/0, 13, 3/0
Histology from biopsy; *n*	HGIN: 8; Invasive SCC: 11	HGIN: 4 Invasive SCC: 8	LGIN: 13 Inflammation: 1 Normal epithelium: 1	LGIN: 10 Inflammation: 3 Necrosis: 1 Normal epithelium: 2
Invasion depth of tumors in resected cases (by ER or SR): *n*	T1a-LPM: 5 T1a-MM: 1 T1b-SM1: 1	T1a-LPM: 5	None	None
Lugol voiding pattern; *n*, A/B/C/D	0/0/2/17	0/1/3/8	0/2/4/9	0/4/5/7
(Proportion of grade D)	(89%)^*∗*^	(67%)^*∗*^	(60%)^*∗∗*^	(44%)^*∗∗*^

SESCC: superficial esophageal squamous cell carcinoma including HGIN; NBI-ME: narrow-band imaging magnifying endoscopy; LCE-PS: Lugol chromoendoscopy with pink-color sign assessment; HGIN: high-grade intraepithelial neoplasia; LGIN: low-grade intraepithelial neoplasia; ER: endoscopic resection; SR: surgical resection; *∗*, *∗∗*: not significant (Pearson's *χ* ^2^ test).

**Table 6:** Comparison of examination times between NBI-ME and LCE-PS (Per-protocol analysis).

Examination time	NBI-ME (*n* = 147)	LCE-PS (*n* = 147)	*P*
Median, s (range)	234 (92–1459)	349 (246–655)	<0.001^*∗*^

NBI-ME: narrow-band imaging magnifying endoscopy; LCE-PS: Lugol chromoendoscopy with pink-color sign assessment; ^*∗*^Mann-Whitney *U* test.
